# Reading a book can change your mind, but only some changes last for a year: food attitude changes in readers of *The Omnivore's Dilemma*

**DOI:** 10.3389/fpsyg.2013.00778

**Published:** 2013-10-31

**Authors:** Julia M. Hormes, Paul Rozin, Melanie C. Green, Katrina Fincher

**Affiliations:** ^1^Department of Psychology, University at Albany, State University of New YorkAlbany, NY, USA; ^2^Department of Psychology, University of PennsylvaniaPhiladelphia, PA, USA; ^3^Department of Psychology, University of North Carolina at Chapel HillChapel Hill, NC, USA

**Keywords:** attitude change, longitudinal, book, narrative, meat

## Abstract

Attitude change is a critical component of health behavior change, but has rarely been studied longitudinally following extensive exposures to persuasive materials such as full-length movies, books, or plays. We examined changes in attitudes related to food production and consumption in college students who had read Michael Pollan's book *The Omnivore's Dilemma* as part of a University-wide reading project. Composite attitudes toward organic foods, local produce, meat, and the quality of the American food supply, as well as opposition to government subsidies, distrust in corporations, and commitment to the environmental movement were significantly and substantially impacted, in comparison to students who had not read the book. Much of the attitude change disappeared after 1 year; however, over the course of 12 months self-reported opposition to government subsidies and belief that the quality of the food supply is declining remained elevated in readers of the book, compared to non-readers. Findings have implications for our understanding of the nature of changes in attitudes to food and eating in response to extensive exposure to coherent and engaging messages targeting health behaviors.

## Introduction

Well-written or compellingly-argued books are often described as “life-changing.” Readers feel that their eyes have been opened to a new way of seeing the world, and they intend to change their behavior to match their new understanding. But to what extent do such books lead to enduring attitude or belief change, vs. having only a temporary impact? How long are individuals' judgments affected by exposure to even an excellent, lengthy, and in-depth persuasive message? The current study provides initial data on this under-explored question.

Individual beliefs and attitudes are of great interest to social, health, and consumer psychologists as they can directly impact behavior. Attitude change is fundamental in accomplishing important social and political changes and in successfully implementing health behavior change. However, studies of attitude change typically only use brief exposures varying from seconds to minutes of recorded material to reading of a few pages of text. These brief exposures are then usually followed by immediate and delayed tests, measured in hours or days, of the effectiveness of the intervention in producing quantifiable attitude change. Only a small number of studies have assessed the duration of experimentally induced attitude change over longer periods of time, such as several weeks, and so far these studies have focused primarily on assessing the extent to which dissonance-provoked or counter-attitudinal change persists over time (Senemeaud and Somat, 2009).

Other studies examine the effect of cumulative exposure to attitude-relevant stimuli. For example, longitudinal studies of the effects of violent media on aggression (Huesmann et al., 2003) and studies of the effects of observing tobacco use in movies on smoking initiation (Heatherton and Sargent, 2009) link repeated exposure to particular types of media with relevant outcomes (e.g., greater aggression after watching more violent television; higher smoking initiation after exposure to movie smoking). Similarly, the cultivation effect (Gerbner et al., 2002) suggests that frequent television viewers show changes in their worldview that match the “mean world” presented on the screen. However, since they focused on the impact of cumulative exposure to multiple stimuli these studies cannot isolate the effects of a particular persuasive message.

An important underexplored area is the impact of experiences such as movies, theater plays, or books, which often transport individuals into a narrative world for hours or even days. Research on narrative persuasion suggests that immersion into narratives can result in attitude, belief, and behavior change (Green and Brock, 2000; Vaughn et al., 2009; Mazzocco et al., 2010; Williams et al., 2011). Narrative persuasion has been demonstrated in domains ranging from health to social and consumer issues (Wang and Calder, 2006; Escalas, 2007; Dunlop et al., 2010). However, much as in other areas of persuasion research, these studies have largely assessed relatively brief stories and short-term outcomes, rather than extended engagement and long-term effects.

Although narratives are sometimes effective because they are perceived as entertaining rather than didactic, other stories or presentations may specifically intend to bring about attitude change. In fact, many widely read books have attitude and behavior change as their explicit aim. Diet books are no exception, and books about eating often integrate compelling personal stories to make their points (e.g., *French Women Don't Get Fat; Animal, Vegetable, Miracle*). Similarly, movies such as *Super-Size Me* (about the negative health effects of fast food) combine entertainment with a clear and persuasive message. There have been some efforts to examine short-term effects of other such presentations, primarily movies, and television programs (Schofield and Pavelchak, 1985; Leiserowitz, 2004; Hether et al., 2008). Surprisingly, though, to the best of our knowledge, no study has assessed the impact on attitudes of controlled exposure to experiences at this level with inclusion of a measure after long delays. The present study systematically investigates the extent and duration of health-related attitude change brought about by exposure to a best-selling book designed to change attitudes to food consumption and the American food supply.

The present study took advantage of an existing program—the University of Pennsylvania Freshman Reading Project—which requests approximately 2000 undergraduate students entering their first year of college to read a designated book prior to their arrival on campus (a different book is assigned each year). A copy of the book is mailed to each entering student during the summer preceding the freshman year. In the 2007/8 academic year the book selected was Michael Pollan's *The Omnivore*'*s Dilemma* (Pollan, 2006), an investigation into the origins of various foods, including organic products, and mass-produced fast food. The book, 464 pages in length, addresses issues such as the modern food production system and food politics in a manner that “promises to change the way we think about the politics and pleasure of eating” (www.michaelpollan.com, 2008). *The Omnivore*'*s Dilemma* was named as one of The New York Times' ten best books of 2006 (“The 10 Best Books of 2006,” 2006), with several factors helping to explain its success, including Pollan's excellent writing and ability to discuss complicated matters in a manner that makes them appealing to a broad audience. One way in which Pollan achieves accessibility and appeal is the high degree of personal involvement that he brings to the book; buying a newborn calf and following its history to slaughter, or working on a chicken killing line at an organic farm. These personal experiences also create emotional involvement and vivid mental images. Pollan chose a topic of current interest, with a general increase in awareness around problems related to the food industry, and growing interest among the public in eating organically and locally.

*The Omnivore's Dilemma* broaches topics of everyday relevance, has numerous implications for the way readers think about the food system, and directly targets personal eating habits. The book is of high quality, as attested to by its bestseller status and critical acclaim. It also includes narrative elements and structure as well as presenting factual information, thus drawing on multiple forms of persuasion. Narrative transportation theory suggests that readers who become immersed in a book are more likely to adopt attitudes and beliefs implied by the story (Green and Brock, 2000), especially if presented with strong and persuasive arguments (Petty and Cacioppo, 1986). Therefore, *The Omnivore's Dilemma* lends itself well to testing the hypothesis that the simple act of reading a book can change the way individuals think and feel. Furthermore, because the book draws on multiple persuasive pathways to reinforce its message, it also provides a powerful test case for examining the long-term persistence of this attitude change.

In addition to the assigned readings, students attended a lecture on the book and participated in group discussions with faculty members, and there were other related events on campus during the school year. This combination of reading and in-person events should also increase the likelihood of long-term persuasive impact. This mix of reading and communal events mirrors entertainment-education efforts across the world. Entertainment-education campaigns embed health messages in an entertaining narrative. A number of these campaigns have supplemented a media presentation related to an issue (e.g., a radio drama; a soap opera) with community efforts such as discussion groups and personal efforts by local health authorities (Singhal et al., 2003).

In sum, the combination of a high-quality book-length message, publicity about the book's themes, and in-person discussions about the book should provide ample opportunity for the formation of strong and enduring attitudes about the book's themes. However, there are also at least two reasons to suspect that attitude change will decrease over time. First, as with any message, the arguments and emotional impact of the book are likely to fade from memory over time. Individuals may forget the content of the book, and the feelings inspired by the book may decline in intensity. Second, attitudes toward food and eating are likely to be linked to ingrained habits and long-held beliefs. Habits are difficult to break (Wood et al., 2002), and therefore individuals may revert back to their prior beliefs as the initial motivation to change fades over time.

Our study had two goals: (1) to examine whether an acclaimed book can produce a substantial change in attitudes related to food production and consumption, and (2), assuming (1), to examine the persistence of attitude change over the course of 1 year.

## Materials and methods

### Participants

This study compared two groups of students enrolled in an introductory psychology course, surveyed at the beginning of two consecutive academic years in 2007 and 2008. In 2007, freshmen in the course were assigned to read *The Omnivore's Dilemma* in the weeks leading up to the beginning of the semester. They were compared to all non-freshmen students in the same course who has not been instructed to read the book in order to gauge the immediate effects of reading the book on attitudes. This first set of participants consisted of 594 undergraduate students. A majority (*n* = 320, 53.9%) were freshmen who had been asked to read *The Omnivore's Dilemma* through the Reading Project. Non-freshmen respondents (primarily sophomores) in the same class were combined into a group of “other” (*n* = 274, 46.1%) and compared to freshman in all analyses, unless otherwise indicated.

In 2008, sophomores in the same introductory course had been assigned to read the book in the previous year and were surveyed in order to examine the extent to which initial attitude change was sustained over the course of 1 year after being exposed to the book's messages. In 2008, readers of the book were once again compared to students who had not been assigned the reading by examining differences between sophomores and non-sophomores enrolled in the course. A total of 567 participants completed the survey in the fall of 2008. This group included 305 freshmen (53.8% of sample), 172 sophomores (30.3%), and 94 others (15.9%). Sophomores were compared to the combined group of non-sophomores in all analyses, unless otherwise indicated. Since students can only enroll in the introductory psychology class once, the 2007 and 2008 samples are non-overlapping.

### Measures

All students in the 2007 class completed an anonymous survey presented through the secure online server Survey Monkey some time between a few days before the start of the fall semester to a few days after the beginning of classes. Respondents indicated basic demographics such as age, gender, and current year in school, along with dietary preferences such as the presence of avoidance of any kind of animal products. They then indicated how much of *The Omnivore*'*s Dilemma* they had read (on a scale of 1–5, where 1 = “I read the entire book,” 2 = “I read most of the book,” 3 = “I read some of the book,” 4 = “I read a little of the book” and 5 = “I did not read the book at all”) and their opinion of the quality of the book (on a scale of 1–5, where 1 = “poor,” 2 = “fair,” 3 = “good,” 4 = “very good” and 5 = “excellent”). Seven questions assessed attitudes to food production and consumption, all related to major thrusts of the book (see Table 1 for exact wording of attitude items). The attitude items were developed by the researchers to capture major themes of the book. Agreement with all attitude items was rated on a 0–10 scale, where 0 = “not at all” and 10 = “extremely or completely.” The attitude items demonstrated acceptable internal consistency (Cronbach's α = 0.74).

**Table 1:** **Attitudes in freshmen who had been assigned The Omnivore's Dilemma in the fall ‘07, compared to non-freshmen'07, and sophomores (i.e., freshmen) 1 year later and non-sophomores in the fall ‘08^a^**.

	**Freshmen‘07 *M (SD)***	**Non-freshmen‘07 *M (SD)***	**Statistic: freshmen vs. non-freshmen ‘07**	**Sopho-mores‘08 *M (SD)***	**Non-sophomores‘08 *M (SD)***	**Sophomores‘07**	**Freshmen‘08**	**Statistic: sophomores vs. non-sophomores‘08**	**Statistic: freshmen'07 vs. sophomores‘08**	**Statistic: freshmen'07 vs. freshmen‘08**	**Statistic: sophomores'07 vs. sophomores‘08**
*n*	320	274		172	399	184	305				
Composite attitude score^*b*^	5.41 (1.75)	4.30 (2.25)	*F*_(1, 307)_ = 8.79, *p* = 0.003, η^2^_*p*_ = 0.03	4.62 (1.68)	4.55 (1.52)	4.40 (2.30)	4.62 (1.52)	*F*_(1, 538)_ = 0.97, *p* = 0.33, η^2^_*p*_ = 0.002	*F*_(1, 428)_ = 17.53, *p* < 0.001, η^2^_*p*_ = 0.04	*F*_(1, 552)_ = 37.93, *p* < 0.001, η^2^_*p*_ = 0.06	*F*_(1, 191)_ = 0.25, *p* = 0.62, η^2^_*p*_ = 0.001
I am reluctant to eat meat	2.54 (3.13)	1.67 (2.42)	*F*_(1, 310)_ = 1.21, *p* = 0.27, η^2^_*p*_ = 0.004	1.95 (3.26)	2.11 (3.19)	1.70 (2.81)	2.15 (3.17)	*F*_(1, 538)_ = 0.002, *p* = 0.96, η^2^_*p*_ < 0.001	*F*_(1, 428)_ = 1.98, *p* = 0.16, η^2^_*p*_ = 0.01	*F*_(1, 552)_ = 3.34, *p* = 0.07, η^2^_*p*_ = 0.01	*F*_(1, 191)_ = 0.05, *p* = 0.82, η^2^_*p*_ < 0.001
I am inclined to buy and eat organic foods	5.42 (3.08)	4.12 (3.42)	*F*_(1, 310)_ = 3.33, *p* = 0.07, η^2^_*p*_ = 0.01	4.51 (3.06)	4.59 (3.20)	4.23 (3.54)	4.75 (3.19)	*F*_(1, 538)_ = 02, *p* = 0.90, η^2^_*p*_ < 0.001	*F*_(1, 428)_ = 6.66, *p* = 0.01, η^2^_*p*_ = 0.02	*F*_(1, 552)_ = 8.03, *p* = 0.01, η^2^_*p*_ = 0.01	*F*_(1, 191)_ = 0.10, *p* = 0.75, η^2^_*p*_ = 0.001
I trust major corporations (reversed)	7.33 (2.25)	6.77 (3.04)	*F*_(1, 310)_ = 1.73, *p* = 0.19, η^2^_*p*_ = 0.01	5.67 (2.44)	5.41 (2.39)	6.90 (2.87)	5.43 (2.40)	*F*_(1, 538)_ = 1.23, *p* = 0.27, η^2^_*p*_ = 0.002	*F*_(1, 428)_ = 50.96, *p* < 0.001, η^2^_*p*_ = 0.11	*F*_(1, 552)_ = 92.08, *p* < 0.001, η^2^_*p*_ = 0.14	*F*_(1, 191)_ = 5.96, *p* = 0.02, η^2^_*p*_ = 0.03
I am inclined to buy and eat local produce	5.80 (2.97)	4.91 (3.53)	*F*_(1, 310)_ = 1.08, *p* = 0.30, η^2^_*p*_ = 0.004	5.20 (2.88)	5.52 (2.69)	5.00 (3.70)	5.69 (2.61)	*F*_(1, 538)_ = 0.95, *p* = 0.33, η^2^_*p*_ = 0.002	*F*_(1, 428)_ = 0.27, *p* = 0.10, η^2^_*p*_ = 0.01	*F*_(1, 552)_ = 0.51, *p* = 0.48, η^2^_*p*_ = 0.001	*F*_(1, 191)_ = 0.05, *p* = 0.83, η^2^_*p*_ < 0.001
I am opposed to government subsidies that promote eating corn	5.04 (2.98)	3.77 (3.43)	*F*_(1, 310)_ = 5.78, *p* = 0.02, η^2^_*p*_ = 0.02	4.11 (2.88)	3.54 (2.67)	3.53 (3.31)	3.49 (2.56)	*F*_(1, 538)_ = 4.61, *p* = 0.03, η^2^_*p*_ = 0.01	*F*_(1, 428)_ = 9.46, *p* = 0.002, η^2^_*p*_ = 0.02	*F*_(1, 552)_ = 4.3.95, *p* < 0.001, η^2^_*p*_ = 0.07	*F*_(1, 191)_ = 0.95, *p* = 0.33, η^2^_*p*_ = 0.01
I am convinced that the quality of the American food supply is declining	6.74 (2.89)	4.79 (3.42)	*F*_(1, 310)_ = 12.47, *p* < 0.001, η^2^_*p*_ = 0.04	5.57 (2.93)	5.03 (2.86)	5.27 (3.43)	5.13 (2.80)	*F*_(1, 538)_ = 5.30, *p* = 0.02, η^2^_*p*_ = 0.01	*F*_(1, 428)_ = 14.48, *p* < 0.001, η^2^_*p*_ = 0.03	*F*_(1, 552)_ = 47.16, *p* < 0.001, η^2^_*p*_ = 0.08	*F*_(1, 191)_ = 0.17, *p* = 0.68, η^2^_*p*_ = 0.001
I am committed to the environmental movement	4.96 (3.00)	4.09 (3.29)	*F*_(1, 310)_ = 1.38, *p* = 0.24, η^2^_*p*_ = 0.004	5.36 (2.55)	5.63 (2.45)	4.17 (3.47)	5.69 (2.42)	*F*_(1, 538)_ = 0.88, *p* = 0.35, η^2^_*p*_ = 0.002	*F*_(1, 428)_ = 3.73, *p* = 0.05, η^2^_*p*_ = 0.01	*F*_(1, 552)_ = 9.16, *p* = 0.003, η^2^_*p*_ = 0.02	*F*_(1, 191)_ = 4.55, *p* = 0.03, η^2^_*p*_ = 0.02

a Agreement with all attitude items was rated on a scale of 0 = “not at all” to 10 = “extremely or completely.”

b Composite Attitude Score was calculated by summing responses on all seven attitude items, with item 3 “I trust major corporations” reverse scored.

In the follow-up to the original study, students in the large introductory psychology class held in the following fall of 2008 completed a brief anonymous online survey during the same time period as the previous year's sample, a few days before or after the beginning of classes. The survey included the same basic demographic information as in the previous year, along with an abbreviated question about the amount of the book read in the previous year (1 = “entire book,” 2 = “some of the book,” and 3 = “none of the book”) and the same seven attitude items rated on the same scale as was done by participants in the previous year (Cronbach's α = 0.68). In the 2008 sample, the sophomores would have been exposed to *The Omnivore's Dilemma* and related campus events 12 months prior, since they were freshmen in the previous year when the book was assigned. The 2008 sample of course also included students in the incoming class of freshmen, who were comparable to freshmen in the previous year in a number of characteristics, including age, proportion of women, and number of red meat avoiders, but importantly differed in the manipulation of interest: unlike the 2007 freshmen, the 2008 freshmen were not sent the book over the summer, were not instructed to read it, and did not experience a set of events early in the semester about *The Omnivore's Dilemma*.

### Statistical analyses

Chi-square tests were used to compare respondents in different class years (e.g., freshmen vs. non-freshmen) on categorical variables, including amount of the book read. A composite attitude score was derived by averaging responses on all seven attitude items (reverse scoring the question about trust in major corporations). In order to explore the overall impact of exposure to the book on attitudes, we conducted a series of univariate analyses of co-variance (ANCOVAs), with the composite attitude score as the dependent variable. We also conducted multivariate analyses of co-variance (MANCOVAs) with all seven attitude items as the dependent variables. Gender was included as a covariate, based on a large body of research suggesting significant gender differences in food-related attitudes and behaviors. These differences include, for example, heightened moral and ecological concerns related to eating, avoidance of seemingly “unhealthy” foods, and a greater focus on health, as opposed to the pleasure of eating in women, compared to men (Rozin et al., 1999; Beardsworth et al., 2002; Wardle et al., 2004). Gender emerged as a significant (*p* = 0.01) covariate in all analyses reported here, with women who read the book generally reporting greater endorsement of the attitudes assessed^1^.

## Results

### Fall 2007: baseline comparison of readers vs. non-readers

As expected, in the fall 2007 class, freshmen were significantly more likely than other students to have read “all or most” (53.6 vs. 0.9% of other respondents) or “some or a little” of the book (40.1 vs. 4.9% of other respondents), as opposed to “none” (6.2 vs. 94.2% of other respondents; χ^2^ = 396.71, *p* < 0.001, Φ = 0.88). Freshmen on average gave a “good” rating of the quality of the book (*M* = 2.93, *SD* = 0.89 on a scale of 1 = “poor” to 5 = “excellent”).

**Figure 1:**
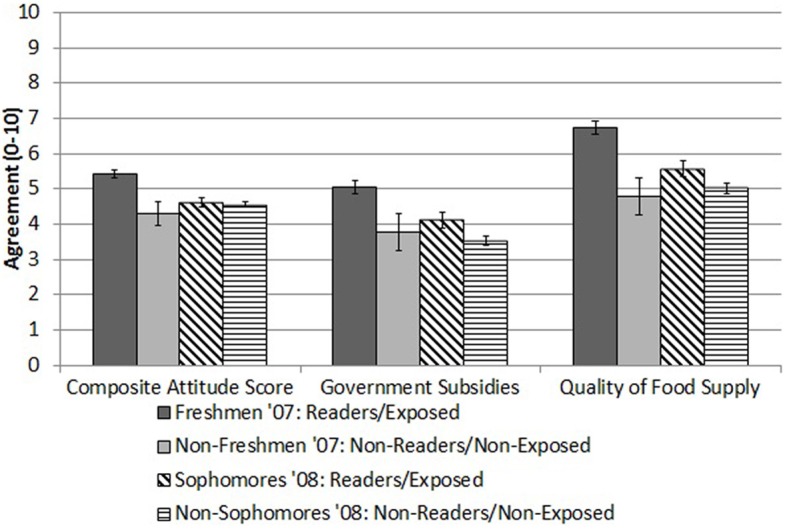
**Composite attitude scores and ratings of opposition to government subsidies and belief that the quality of the food supply is decreasing (*M, SE*) in students assigned The Omnivore's Dilemma (“exposed”) vs. not reading the book (“unexposed”) in 2007 (Freshmen vs. Non-Freshmen) vs. 2008 (Sophomores vs. Non-Sophomores)**.

As predicted there was a significant main effect of class year (i.e., freshmen vs. non-freshmen, that is, readers and non-readers) on the composite attitude score (Table 1). Freshmen overall reported significantly greater agreement, compared to non-freshmen, with a mean difference of about one unit (*M* = 1.10, *SD* = 0.36 on a scale of 0–10) on the composite attitude score (Table 1, Figure 1). There was a significant multivariate main effect of class year on combined ratings of the seven individual attitude items [*F*_(7, 301)_ = 2.25, *p* = 0.03, *Wilk*'*s lambda* = 0.95, η^2^_*p*_ = 0.05]. Significant differences between freshmen and non-freshmen emerged on two of the seven attitude items. Freshmen reported significantly greater opposition to government subsidies for corn production and more agreement with the statement that the quality of the American food supply is declining (both *p* < 0.05; Table 1, Figure 1). There were significant positive relationships among freshmen between composite attitude scores and amount of the book read (*r* = 0.30, *p* < 0.001) as well as ratings of the quality of the book (*r* = 0.34, *p* < 0.001).

### Fall 2008: comparison of readers vs. non-readers at one-year follow-up

As was expected, in the fall 2008 class, sophomores (readers) were significantly more likely to have read *The Omnivore's Dilemma*, compared to the combined group of other students, with 82.4% (*n* = 136) indicating having read “some or all” of the book, compared to 3.9% (*n* = 15) of other students (χ^2^ = 356.83, *p* < 0.001, Φ = 0.81). While 2008 sophomores were not asked about their liking of the book, we did replicate the finding from the previous year of a significant positive relationship between the amount of the book read and composite attitude scores (*r* = 0.23, *p* = 0.004). In the 2008 class there was no statistically significant effect of class year (i.e., sophomores vs. non-sophomores) on the composite attitude score [*F*_(1, 538)_ = 0.97, *p* = 0.33, η^2^_*p*_ = 0.002; Table 1]. Mean composite attitude scores were almost identical in the 2008 sophomores and the 2008 non-sophomores (*M* = 4.62 vs. *M* = 4.55; Table 1, Figure 1). There was no statistically significant multivariate effect of class year on the combined attitude items [*F*_(7, 532)_ = 1.89, *p* = 0.07, *Wilk*'*s lambda* = 0.98, η^2^_*p*_ = 0.02], though *post-hoc* analyses revealed that sophomores differed significantly from other students in the 2008 class in their responses to two of the seven attitude items, reporting more opposition to government subsidies for corn production and a greater belief that the quality of the American food supply is diminishing (Table 1, Figure 1). Interestingly, though statistically not significant, sophomores' agreement with four of the other five attitude items was on average *lower* than that of their classmates who had never been assigned the reading.

### Readers of *The Omnivore's Dilemma* in 2007 and 2008

We next compared sophomores in the 2008 class who had previously been exposed to the book to freshmen who had been assigned the book in 2007. This analysis compares members of the same cohort measured 1 year apart (i.e., two non-overlapping groups in which the vast majority of individuals read at least a little of *The Omnivore's Dilemma*). There was a significant difference in composite attitudes scores, with sophomores reporting overall less agreement, compared to freshmen in the previous year (Table 1, Figure 1). Similarly, there was a significant multivariate main effect of class year on the combined attitude ratings [*F*_(7, 422)_ = 11.79, *p* < 0.001, *Wilk*'*s lambda* = 0.84, η^2^_*p*_ = 0.16]. Significant differences emerged on all but two of the attitude items (Table 1). With the exception of their commitment to the environmental movement (which increased significantly), reluctance to eat meat, and inclination to buy local foods (which remained unchanged), agreement with individual attitude items and the composite attitude score dropped significantly over the course of 12 months after having read the book (Table 1). In fact, sophomores in 2008, all of whom had been assigned the book in the previous year, looked quite similar to sophomores in 2007, almost all of whom had not read the book, in terms of composite attitude scores [*F*_(1, 191)_ = 0.25, *p* = 0.62, η^2^_*p*_ = 0.001] and ratings of all but two attitude items [*F*_(7, 185)_ = 2.02, *p* = 0.05, *Wilk*'*s lambda* = 0.93, η^2^_*p*_ = 0.07] (Table 1).

### Freshmen in 2007 vs. 2008

Finally, we compared freshmen in 2007 to freshmen in 2008 as another way of isolating the initial effects of the book from other influences on attitudes that may generally be present as students transition into the new campus environment. Compared to freshmen who were assigned the book in 2007, freshmen in 2008 (who had not been assigned the reading) indicated significantly lower composite attitude scores (Table 1, Figure 1) and significantly lower agreement with the set of individual attitude items [*F*_(7, 546)_ = 25.61, *p* =< 0.001, *Wilk*'*s lambda* = 0.75, η^2^_*p*_ = 0.25], with significant differences emerging on all but two items (reluctance to eat meat and inclination to purchase local foods). Freshmen in 2007 indicated greater agreement with all these items, with the exception of their commitment to the environmental movement, which was greater in freshmen in 2008 (Table 1).

Of note, 6.2% (*n* = 18) of freshmen in 2007 and 17.6% (*n* = 29) of sophomores in 2008 indicated not having read any of the book. None of the findings reported differed substantially based on whether or not these students were excluded from the analyses. This may in part be due to the fact that while they may not have read the book, these students were nonetheless exposed to its content through related campus events, especially during the week before the onset of the school year, as well as in discussions with other members of their class. Therefore, results for the entire sample—including the minority of individuals who were assigned but did not read the book—are presented here.

## Discussion

Reading *The Omnivore's Dilemma* had a substantial short-term impact on overall attitudes related to food production and consumption, as indicated by significant differences in composite attitude scores between freshmen who had read the book and non-freshmen who had not been assigned the reading. Specifically, attitudes about the perceived quality of the food supply and toward government subsidies for corn production were impacted in the predicted direction.

Attitude change dissipated somewhat with time, as reflected in non-significant differences in composite attitude scores between sophomores (who had read the book) and non-sophomores (who had not read the book) the following year, as well as significant differences between freshmen in year 1 and sophomores in the following year (all of whom had read the book, but at different time points). Self-reported opposition to government subsidies and belief that the quality of the food supply is declining remained elevated in readers of the book over the course of 12 months, as illustrated by significant differences between sophomores and non-sophomores in 2008; however, there was some decrease in reported agreement with these statements after 1 year, as reflected in significant differences between freshmen in year 1, and sophomores in the following year, all of whom had been assigned to read the book.

To the best of our knowledge, this is the first time the impact of an extended stimulus such as a book on health-related attitude change has been assessed systematically, and tracked over an extended period of time. Identifying patterns of attitude persistence and the dissipation of influence has important implications for our understanding of the mechanisms underlying health behavior change, including the impact and effectiveness of exposures to interventions targeting attitudes at a comparable level, including movies or plays.

The overall attitude composite declined substantially, and even the two beliefs that demonstrated continued change showed some dissipation over the course of a year. Although our study did not directly test the cognitive processes underlying this pattern of attitude change, there are two potential theoretical reasons why this effect occurred. The first is simple forgetting; memory for the content of messages declines quickly. Although attitude change does not necessarily depend on message recall (Greenwald, 1968), it is likely that over an extended time, participants' own cognitive and emotional responses to the book also faded from memory, which would lead them to revert to their prior attitudes. The second probable mechanism for the decline in the overall attitude composite is the difficulty of changing eating behavior and health habits in general (Wood et al., 2002). Individuals might have been temporarily motivated to engage in some of the behaviors captured by the composite attitude score, including buying local/organic food or giving up eating meat, but such goals may have been competing against existing strong habits. Furthermore, as noted previously, if individuals were not able to break those habits, they may have unconsciously reverted to their previous attitudes in order to reduce dissonance.

The attitudes that did show enduring change were clearly demonstrated in the book and were likely relatively new for participants (e.g., corn subsidies and food quality are not typical topics of discussion among high school and college students, and these issues may receive infrequent news coverage). Furthermore, endorsement of these beliefs did not imply specific individual-level behavior changes, so they were unlikely to conflict with existing habits.

Future studies should investigate factors which distinguish between attitude change that endures and that which fades, as well as considering whether there may be particular subsets of individuals who do experience enduring change on a wider variety of attitudes and behaviors. For example, individuals who were especially transported into the book or those who judged it as “excellent” might be more likely to maintain long-term attitude change, as might those who are more easily transported in general (Dal Cin et al., 2004; Mazzocco et al., 2010). Individuals high in need for cognition who viewed the arguments as strong may also have been more likely to form enduring attitudes (Petty et al., 1995). Because our current design did not include individual difference measures or assess the same individuals at multiple time points regarding their liking of the book or amount read, it did not allow us to test these possibilities. Therefore, repeated-measures longitudinal studies addressing these processes are an important next step in investigating attitude change over extended time periods.

Furthermore, future research should examine whether the findings presented here may extend to attitude change as assessed using standardized measures of food-related attitudes, along with indicators of actual behavior change that may result from changes in attitudes (e.g., an increase in purchases of organic foods, decrease in meat consumption). This appears especially relevant in light of recent findings to suggest that in young adults, more positive attitudes toward organic, local and sustainable foods are associated with the consumption of a diet of significantly higher quality (Pelletier et al., 2013). More research is also needed to disentangle the impact on readers' attitudes of different ways in which the message of the book was transmitted in the present study. Here, participants were not only assigned the reading but also participated in events designed to have them engage further with the content of the book. The effects of the reading itself vs. being exposed to the book's messages via other channels therefore could not be isolated. Future studies should also consider including a control group that is exposed to the message of a book, but without having it be embedded in a narrative in order to examine the effects of narrative transportation vs. simple exposure to the message itself.

One alternative explanation for our results is that the reported initial attitude change was not true attitude change, but simply a reporting bias due to social pressure: students may have felt that they were expected to accept the conclusions of the book because it was assigned as University reading. However, there are several reasons why this explanation is implausible. First, students completed the survey online using only their student ID. Second, students in the survey were willing to admit that they had not read the entire book, and they also gave a wide range of ratings of the quality of the book. Finally, examination of the means and standard deviations for the attitude items suggests that there was substantial variation in responding, which speaks against strong social desirability pressures.

It can be concluded that the simple act of reading a book can have a significant impact on attitudes related to food production and consumption. However, it appears that at least some of this attitude change is relatively short in duration, with effects declining or disappearing over the course of a year. Of course, in many circumstances, major exposure to a point of view or a particular event, is reinforced over and over again in subsequent weeks, months, or even years. The extent to which the effects reported here generalize from eating behavior to other health habits, and from the book studied here to other publications and comparable stimuli such as movies or television programs should be assessed in future studies. It also remains to be determined whether this pattern of selective longer-term impact extends to fiction or other works of literature (Appel and Richter, 2007). Based on findings presented here it might also be possible, under experimental conditions, to alter the content of a book or movie, to assess the importance of various components of the intervention that may contribute to attitude change.

### Conflict of interest statement

The authors declare that the research was conducted in the absence of any commercial or financial relationships that could be construed as a potential conflict of interest.

## Footnotes

^1^We hypothesized that vegetarian status (i.e., self-reported avoidance of any animal products such as red meat, poultry, fish, dairy, and/or eggs) may also have impacted reported attitudes due to potentially greater awareness of issues related to food production, quality, and sustainability in those choosing to avoid meat and other animal products. We repeated all analyses reported here with vegetarian status included as a covariate and while it emerged as statistically significant in all comparisons, it did not otherwise impact results, with the single exception of the difference between Freshmen in'07 and Freshmen in'08 in their reported reluctance to eat meat, which was no longer statistically significant with the inclusion of the covariate. All results reported here are based on analyses that did not include vegetarian status as a covariate.
